# The pearl necklace model in protein A chromatography: Molecular mechanisms at the resin interface

**DOI:** 10.1002/bit.26843

**Published:** 2018-10-25

**Authors:** Goncalo L. Silva, Jacek Plewka, Helga Lichtenegger, Ana C. Dias‐Cabral, Alois Jungbauer, Rupert Tscheließnig

**Affiliations:** ^1^ CICS‐UBI – Health Sciences Research Centre, University of Beira Interior Covilhã Portugal; ^2^ Department of Chemistry University of Beira Interior Covilhã Portugal; ^3^ Department of Biotechnology, Austrian Centre of Industrial Biotechnology Vienna Austria; ^4^ Department of Material Science and Process Engineering University of Natural Resources and Life Sciences Vienna Austria; ^5^ Department of Biotechnology University of Natural Resources and Life Sciences Vienna Austria

**Keywords:** affinity chromatography, monoclonal antibodies, pearl necklace model, radial density distribution, small‐angle X‐ray scattering, staphylococcal protein A

## Abstract

Staphylococcal protein A chromatography is an established core technology for monoclonal antibody purification and capture in the downstream processing. MabSelect SuRe involves a tetrameric chain of a recombinant form of the B domain of staphylococcal protein A, called the Z‐domain. Little is known about the stoichiometry, binding orientation, or preferred binding. We analyzed small‐angle X‐ray scattering data of the antibody–protein A complex immobilized in an industrial highly relevant chromatographic resin at different antibody concentrations. From scattering data, we computed the normalized radial density distributions. We designed three‐dimensional (3D) models with protein data bank crystallographic structures of an IgG1 (the isoform of trastuzumab, used here; Protein Data Bank: 1HZH) and the staphylococcal protein A B domain (the native form of the recombinant structure contained in MabSelect SuRe resin; Protein Data Bank: 1BDD). We computed different binding conformations for different antibody to protein A stoichiometries (1:1, 2:1, and 3:1) and compared the normalized radial density distributions computed from 3D models with those obtained from the experimental data. In the linear range of the isotherm we favor a 1:1 ratio, with the antibody binding to the outer domains in the protein A chain at very low and high concentrations. In the saturation region, a 2:1 ratio is more likely to occur. A 3:1 stoichiometry is excluded because of steric effects.

## 1 INTRODUCTION

Staphylococcal protein A chromatography is the capture step of choice in the manufacturing of monoclonal antibodies because of its high selectivity and robustness (Hahn et al., 2005; Hahn, Shimahara, Steindl, & Jungbauer, 2006; Shukla, Hubbard, Tressel, Guhan, & Low, 2007). *Staphylococcus aureus* protein A is a cell wall 56‐kDa protein with five homologous binding domains, designated as E, D, A, B, and C, in order from the N‐terminal (Ghose, Allen, Hubbard, Brooks, & Cramer, 2005; Graille et al., 2000; Hober, Nord, & Linhult, 2007; Starovasnik, O’connell, Fairbrother, & Kelley, 1999; Uhlén et al., 1984). MabSelect SuRe (GE Healthcare) is one of the most widely used protein A resins. It has a tetrameric chain of synthetically engineered Z‐domains, which are derived from the B‐domain with point mutations to improve alkaline stability (Ghose et al., 2005).

Protein A binding to immunoglobulin G (IgG) occurs through the hydrophobic region between the CH_2_ and CH_3_ domains of the Fc, known as consensus binding site (Deisenhofer, 1981; DeLano, Ultsch, de Vos, & Wells, 2000; Gagnon, Nian, Leong, & Hoi, 2015; Salvalaglio, Zamolo, Busini, Moscatelli, & Cavallotti, 2009; Shukla et al., 2007). Despite having physical–chemical properties that make it prone to establishing hydrogen bonds and electrostatic interactions, it is because of its exposed hydrophobic moiety, the consensus binding site shows preferential binding with the protein A ligands (Salvalaglio et al., 2009). Irrespective of the abundant information regarding Fc recognition by protein A, antibody structural rearrangement upon adsorption to protein A ligands and the associated stoichiometry are not fully understood. However, some authors have reported the possibility of multiple binding to protein A chains, but with protein A in solution (Ghose, Hubbard, & Cramer, 2007). Others have also addressed this issue, reporting the possible antibody binding orientations of an IgG4 to immobilized protein A in silica (Mazzer et al., 2017).

Molecular models have been applied to study antibody form and flexibility in aqueous solutions (Brandt, Patapoff, & Aragon, 2010; Sandin, Öfverstedt, Wikström, Wrange, & Skoglund, 2004) for a better understanding of antibody aggregate adsorption to protein A resins (Yu et al., 2016) and to characterize the nature of antibody binding to protein A (Salvalaglio et al., 2009; Zamolo, Busini, Moiani, Moscatelli, & Cavallotti, 2008). Salvalaglio et al. (2009) and Zamolo et al. (2008) have described that regions and amino acids play a major role in the interaction with chromatography matrices based on the crystal structure of CH_2_ and CH_3_ of an IgG1 coupled with fragment B of protein A determined by Deisenhofer (1981) (PDB: 1FC2). However, despite this high economic value, a real three‐dimensional (3D) structure of the antibody–staphylococcal protein A complex based on experimental data at different antibody loadings has not been elucidated. The current state‐of‐the‐art on antibody–protein A conformations is solely attributed to the computational simulations (Busini, Moiani, Moscatelli, Zamolo, & Cavallotti, 2006; Salvalaglio et al., 2009).

Here we presented a methodology capable to experimentally assess normalized radial densities of antibody–protein A conformations at a resin surface by small‐angle X‐ray scattering (SAXS). SAXS provided information at the structural level of particle systems of the colloidal size (to thousands of angstroms, Å), such as antibodies (Boldon, Laliberte, & Liu, 2015). SAXS is based on the concept that a particle of relatively greater size than the X‐ray wavelength will scatter the incident X‐ray. On the basis of the scattering intensity, it is possible to assess form, shape, and size of the scatterer. Therefore, it would be possible to establish an approximation of the “spatial extension of the particle”. SAXS can provide information from a dynamic system and take into account molecular flexibility and different configurations (Boldon et al., 2015).

In this work we investigated the adsorption of a monoclonal antibody to MabSelect SuRe. More concisely, we sought to obtain an overview of the structural rearrangement of the antibodies in the tetrameric protein A and to estimate the evolution of surface layer thickness with antibody concentration, as well as the antibody–ligand stoichiometry. We compared the antibody–protein A complex radial densities provided by SAXS with theoretical configurations (protein AB domain from the crystal structure 1BDD and the antibody from the crystal structure 1HZH from Protein Data Bank [PDB]) and spatial rearrangement of antibodies and staphylococcal Protein A ligands using a molecular model approach. We implemented this model to simulate different binding orientations of a crystallographic structure of an IgG1 to a tetrameric B‐domain protein A chain attached to an agarose structure to mimic the experimental system of a monoclonal antibody to MabSelect SuRe. In the current study, the methodology is explored on this very specific system of high industrial relevance, but it is also applicable to a broad range of protein‐surface adsorption systems and can improve the understanding of protein binding in those systems.

## 2 MATERIALS AND METHODS

### 2.1 Materials

Trastuzumab was purchased from Roche (Basel, Switzerland; Lot B1050B07). All the following reagents were purchased from MilliporeSigma (Burlington, MA): sodium phosphate dibasic (Na_2_HPO·2H_2_O; Lot K450726804049), sodium dihydrogen phosphate (NaH_2_PO·2H_2_O; Lot K93717142706), glycine (C_2_H_5_NO_2_; Lot VP614601407), and sodium chloride (NaCl; Lot K48705904713). MabSelect SuRe resin was purchased from GE Healthcare (Uppsala, Sweden; Lot 10247535).

### 2.2 Adsorption isotherms

The antibody solutions were prepared in 0.02 M phosphate buffer with 0.15 M sodium chloride at pH 7.4 in a range from 0.01 to 10 mg/ml. A volume of 0.025 ml of resin was added to the antibody solution with a total volume of 0.25 ml. The samples were incubated for 24 hr in a thermomixer (Thermo Fisher Scientific, Waltham, MA) at 20°C and 900 rpm. After incubation, the bulk concentration was measured at Abs 280 nm using a UV plate reader (Tecan, Männedorf, Switzerland).

### 2.3 Scanning electron microscopy

The MabSelect SuRe beads were first submerged in a cryoprotectant 2.3 M sucrose solution. The sample was then frozen with liquid nitrogen and the beads were cut into slices 30‐µm thick using a tungsten carbide knife in an MT‐990 Motorized Precision Microtome (RMC Boeckeler). The bead slices were dehydrated with ethanol series and then dried with CO_2_ in a Critical Point Dryer Leica EM CPD030. For the visualization, we used a Scanning Electron Microscope Quanta™ 250 FEG, and the dried slices were placed on an aluminum slab and coated with a gold layer.

### 2.4 SAXS

The SAXS measurements were performed in the beamlines BM26B (Portale et al., 2013) and BM29 (Pernot et al., 2013) at the European Synchrotron Radiation Facility (Grenoble, France). The antibody sample preparation followed the same procedure as for the adsorption isotherm measurements. After the incubation, the solution was resuspended, and 100 µL of incubated sample was loaded into a quartz capillary. The capillary was then placed aligned to the beam. The scattering images were collected in 10 frames at 1‐s exposure each using Pilatus 1 M detector at 12 keV (*λ* = 1.033 Å).

### 2.5 Modeling

SAXS is a powerful and effective technique for determining molecule shapes and sizes at the nanoscale length. This approach measures the scattering intensity I(Q) function of a scattering vector Q resulting from a scattering angle 2θ, at a given wavelength *λ*, where Q=4πsinθ/λ. Q values are correlated to real‐space distances d with d=2π/Q (Hayter & Penfold, 1983; Zhang et al., 2007).

### 2.6 A fractal pearl necklace model

The antibody binds to protein A ligands and a complex is formed. This complex could be described by its characteristic pair density distribution. The Fourier transform of the pair density distribution gives the form factor, P(Q), which is the scattering intensity of the complex according to its characteristics, such as shape, size, or concentration. In addition, pair density distributions of complexes randomly arranged in the fractal network of the agarose resin contribute to the structure factor, S(Q), and can be described by $p_{S}(r)\propto r^{D_{f}}\text{exp}(-\kappa r)$. Under the assumptions of the scattering theory, the scattering intensity of the whole system is not more than the product of the form and structure factor: I(Q)=P(Q)S(Q). The scattering intensity curve is obtained by

(1) $$I(Q)=ℱ(p(r))[Q]={\int^{\;}}_{0}^{\infty }drp_{P}(r)\;J_{1/2}(Qr)/{\left(Qr\right)}^{1/2}Q^{-(D_{f}+1)},$$

where $J_{1/2}$ is a Bessel function of the first kind of order ½. The form factor is the Fourier transform of the radial density distribution: $P\left(Q\right)=ℱ\left(p_{p}\left(r\right)\right)={\int^{\;}}_{0}^{\infty }drp_{p}\left(r\right)\;J_{1/2}(Qr)/{\left(Qr\right)}^{1/2}$. The structure factor is the Fourier transform of the pair density distribution of the fractal network: $S\left(Q\right)=ℱ\left(p_{S}\left(r\right)\right)\propto Q^{-\left(D_{f}+1\right)}$.

It is challenging to normalize any scattering intensity. The scattering intensity depends on the chemical contrast of each entity and may decrease despite the increasing number of scatterers. We shift the normalization issue to real space. We introduce the normalized pair density distribution of spherical hulls $p_{p}\left(r,R\right)\propto r/R^{2}H(2R-r)$ and hereby enforce radial symmetry. It is an essential step that solves the normalization problem in a very elegant way. We define our working equation as

(2) $$I\left(Q\right)Q^{\left(D_{f}+1\right)}\propto {\int^{\;}}_{0}^{\infty }dR4\pi R^{2}p'(R)|\;J_{1/2}(QR)/{\left(QR\right)}^{1/2}{|}^{2}.$$

The variable R is the measured distance from the scattering site relative to the backbone of the agarose. This mathematical model resembles a fractal folded pearl necklace, made from pearls with an average radial density distribution of matter, p′(R).

### 2.7 The fractal network of the resin imposes a fractal structure factor, *S*(*Q*)

In the present case, we monitor antibody adsorption at high concentrations. Thus, the antibody concentration in the proximity of the surface is high. This is the reason why the infinite dilution argument no longer holds true. We have to take into account complex–complex pair density distributions. It seems appropriate to characterize their structure by the fractal pair density distribution: $p_{S}\left(r\right)\propto r^{D_{f}}\text{exp}(-\kappa r)$, with κ as the screening length. Then, the structure factor of protein–ligand complexes resembles:

(3) $$S(Q)=ℱ(p_{S}\left(r\right))[Q]={\int^{\;}}_{0}^{\infty }p_{S}\left(r\right)\;J_{1/2}(Qr)/{\left(Qr\right)}^{1/2}.$$

It is a sin‐transform of the fractal pair density distribution:

(4) $$ℱ(p(r))[Q]=C_{D_{f}}/{\left(\kappa^{2}+Q^{2}\right)}^{(D_{f}+1)/2}\sin\left((D_{f}+1)\tan^{-1}\left(\frac{Q}{\kappa }\right)\right),$$

where $C_{D_{f}}$ is a proportional constant of the gamma function Γ: $C_{D_{f}}=\sqrt{\left(2/\pi \right)}\left(\Gamma (D_{f}+2)/\kappa^{D_{f}+1}D_{f}\;+\;1\right)$. In the case of infinitely small κ, it simplifies to: $ℱ(p(r))[Q]\propto 1/Q^{D_{f}+1}$.

### 2.8 Bi‐Langmuir adsorption

MabSelect SuRe is known for its tetrameric chain of B‐domain‐derived ligands. These four theoretical antibody binding domains may be a source of energetic heterogeneity. Therefore, the Langmuir isotherm may incorrectly predict adsorption for this system. High energy adsorption sites become saturated (i.e., are occupied first) at low concentrations, while at high concentrations, molecules adsorb to high and low energy sites (Gritti & Guiochon, 2010). The system is better described by a bi‐Langmuir model, which takes into account this possible heterogeneous adsorption as it is based on the coexistence of two (or more) independent noncooperative sites (Bellot & Condoret, 1993; Gritti & Guiochon, 2005). The amount of adsorbed protein q in equilibrium with equilibrium solution concentration *C* is modeled by

(5) $$q=\overset{2}{\underset{i=1}{\sum^{\;}}}q_{i,m}b_{i}C/(1+b_{i}C),$$

where $q_{i,m}$ gives the maximum adsorbed capacity at any site, and $b_{i}$ values are the sample equilibrium constants between the bulk solution and the multiple adsorption sites and $b_{i}>0$.

## 3 RESULTS AND DISCUSSION

The scope of this study is to understand the rearrangement and orientation of antibodies on MabSelect SuRe. SAXS is the fingerprint technique used here, and antibody–protein A interaction data interpretation was done in terms of radial density distribution. We computed hypothetical 3D models and thereof radial density distributions. We compared the results to radial density distributions we computed from experimental scattering data. We found favored binding orientations and stoichiometry. Scanning electron microscopy (SEM) imaging was used to validate the determination of the structure factor of a defined fractal network composed by the resin’s cross‐linked agarose.

### 3.1 Determination of the antibody–protein A 3D complex and its structural rearrangement

#### 3.1.1 Scattering profiles

**Figure 1:**
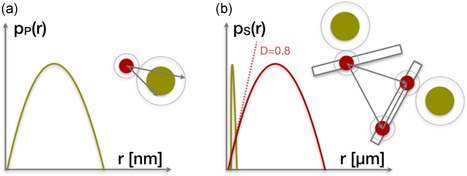
Schematic representation of the antibody (green disks) complexed with the protein A ligand (red disks), their distribution across the resin network (gray rectangles), and the respective pair density distributions for form and structure factors. (a) The green line is the hypothetical pair density distribution $p_{p}\left(r\right)$ of the antibody and the ligand; (b) the hypothetical pair density distribution, $p_{s}\left(r\right)$, of the agarose is presented by the red line. The hypothetical $p_{p}\left(r\right)$ is superimposed by a green line for scale. The slope of the tangent at small *r* to $p_{s}\left(r\right)$ is the fractal dimension Df of the agarose network [Color figure can be viewed at wileyonlinelibrary.com]

The SAXS data were analyzed according to the mathematical framework drafted in the theory section and outlined in Figure 1. In the current study, we assumed that the scattering intensities could be split into a product of form and structure factors. This simplification was made because of the different scales of the radial density distribution of both the antibody–protein A ligand complex and the distribution of these particular complexes in the resin. The form factor, P(Q), computed from the radial density distribution, mimics the statistics of the distances measured within the antibody–protein A ligand complex, with a typical distribution as depicted in Figure 1a. The red disk is a schematic representation of the protein A ligand; the larger green disk mimics the immobilized antibody. The structure factor, S(Q), takes into account the distribution of these complexes throughout the resin network, with a possible arrangement shown in red in Figure 1b. We assume a random distribution of ligands, and it is the particular structure of the resin that imposes the characteristic shape of the pair density distribution from which the antibody–ligand complex structure factor is computed.

#### 3.1.2 Scanning electron microscoe (SEM)

Parallel to SAXS data, we used SEM to visualize the agarose beads of MabSelect SuRe. From the SEM image, we could computationally generate the structure factor of the fractal network and compare it with the obtained value from SAXS.

**Figure 2:**
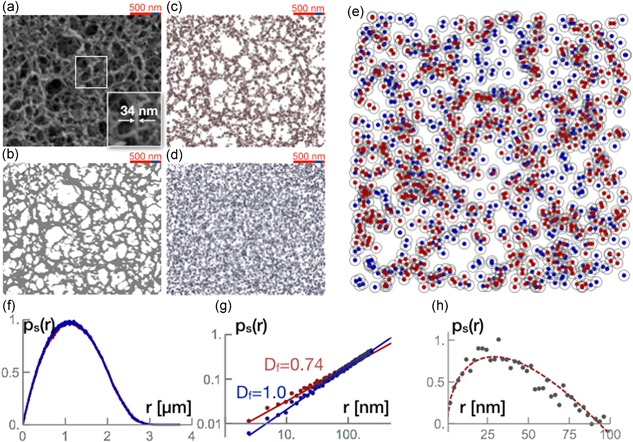
(a) SEM image of MabSelect SuRe resin. The red scale bar indicates a 500 nm distance. The insert is a magnification of a typical agarose strand. Strands are up to 34 nm in diameter. (b) Binarized SEM image. The pores are identified as white areas and the agarose as gray. (c) Random choice of 10,000 pixels distributed across the agarose fractal network (red). (d) Random choice of 10,000 pixels of SEM image (agarose fractal network and the pores—white noise; blue). (e) Magnification of the overlay of (c) and (d), where the red dots represent the random distribution of the agarose, and the blue dots the random distribution of the agarose and the pores. (f) Pair density distribution of both the fractal network (red) and white noise (blue). (g) Determination of the dimension of the fractal network (Df = 0.74) and white noise (Df = 1.02). (h) Subtraction of the pair density distribution of the fractal network and white noise: pore size distribution, with the largest being 80 nm [Color figure can be viewed at wileyonlinelibrary.com]

Figure 2a shows a SEM image of MabSelect SuRe resin’s network. The magnification indicate a typical diameter of approx. 34 nm. The SEM image was binarized, resulting in Figure 2b, where gray areas indicated the agarose network and white areas mark the pores. From the binarized image, we chose 10,000 sites randomly distributed in two zones. First, we constrained the site choices to the gray areas, that is, the agarose network, and displayed them with red dots in Figure 2c. Then, we randomly chose pixels from both the gray and the white areas (random noise over the whole picture), marking them with blue dots in Figure 2d; these are considered white noise. Figure 2e is a magnified overlay of Figure 2c,d. From Figure 2e, we computed the normalized pair density distribution to the same amount of relative distances from the red and blue sites and plotted them in Figure 2f. The pair density distribution can be estimated with $p_{S}(r)=r^{D_{f}}$. Therefore, from these pair density distributions, we computed the dimensionality $D_{f}$ of the system. Whereas the white noise data gave a value $D_{f}=1.0$, the fractal dimension of the agarose network returned a value $D_{f}=0.74$, both represented as the slope of the fit curve to the data in Figure 2g. The pair density fluctuations were determined with $\Delta p_{S}(r)=r^{0.74}-r^{1.0}$. It is an approach to estimate the average pore form and average pore sizes at a small scale. Figure 2h shows the pair density fluctuations of the MabSelect SuRe resin as assessed by the SEM image, with the typical small pores close to 80 nm in diameter (Pabst, Thai, & Hunter, 2018).

#### 3.1.3 Antibody solution

**Figure 3:**
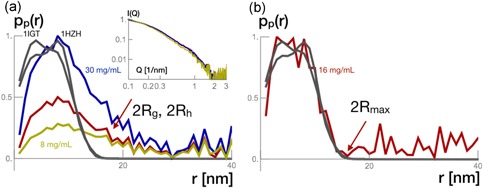
(a) Normalized scattering intensity (insert) from antibody in solution at 8 (green), 16 (red), and 30 mg/ml (blue). The respective pair density distributions plotted with the pair density distribution from the crystallographic structures 1HZH and 1IGT. (b) Overlay of the pair density distribution from the crystallographic structures 1HZH and 1IGT with the pair density distribution of the subtracted scattering intensities from the antibody in solution at 16 and 8 mg/ml [Color figure can be viewed at wileyonlinelibrary.com]

To appropriately describe the adsorption mechanism, it is essential to evaluate the antibody state of aggregation at the used solution concentrations. The form factor of the antibody was computed from measurements of antibody in solutions at 8, 16, and 30 mg/ml. Figure 3a shows in the insert the scattering intensity curves from the antibody in solution samples and the respective pair density distribution, as well as the pair density distribution of two antibody crystallographic structures (PDB: 1IGT—IgG2, and PDB: 1HZH—IgG1) to complement SAXS data evaluation.

The pair density distributions from the crystallographic structures were computed from the centroids of the amino acids. They present maxima at 3 and 7 nm values, which in literature are associated to the hydrodynamic radius of an IgG1 and would match well with the experimental data for the antibody in solution (Gagnon et al., 2015). Pair densities could be found up to relative distances of 12 nm. Indeed, this value is an estimation of the hydrodynamic diameter of the antibody in solution considering all the associated intrinsic flexibility (Gagnon & Nian, 2016; Gagnon et al., 2015).

The relative pair density $p_{P}\left(r\right)$ from the experimental samples a tailing profile up to relative distances larger than the antibody size. This behavior may be characteristic of pairwise interactions between molecules in solution that are near each other and consequently, these additional relative distances contribute to the scattering signal. It would be as well valid to accuse a certain biologic flexibility of the antibody molecules in solution (Boldon et al., 2015), opposed to their rigid structures in the crystals. Indeed, one of the advantages of SAXS over crystallographic data in terms of distance assessment measurements relies on the fact that the scattering intensity of a particle is measured in solution. Both interpretations, the pairwise interactions of antibody molecules and the flexibility of a monomer, are attributed similarly to the scattering intensity. Both contributions could be addressed by a factor $\propto Q^{D}$. The physical interpretation of the exponent D differs though. First, in the case of pairwise interactions, it would be the parameter of an interaction potential. Second, it would be interpreted as a measure of size distributions of the protein taking into account the intrinsic biological flexibility of the monomer. Both interpretations impacted the possible adsorption process.

We suggest a different evaluation. To assess the pair density distributions of the monomeric form, we subtracted the appropriately normalized pair densities from the sample at solution concentration 8 mg/ml from that at solution concentration 16 mg/ml. The signal is plotted in Figure 3b along with the pair density from the crystallographic structures. The results match the crystallographic data and we may argue that up to 16 mg/ml protein solutions are monomeric. Consequently, we anticipate, that antibody monomers adsorb and that the tailings in pair densities are due to parasitic background. For 30 mg/ml, we indeed do monitor a factor of $Q^{-0.3}$. However, 16 mg/ml is well above the antibody starting concentration at which we perform our measurements.

#### 3.1.4 The structure factor

**Figure 4:**
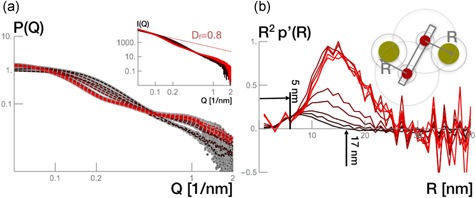
(a) The scattering intensity, P(Q), given as a function of the scattering vector, Q (1/nm), from antibody bound to MabSelect SuRe 0–80 mg/ml resin by gray disks. Fits are represented in black and evolve toward red with increasing antibody concentration. Insert shows the raw experimental datasets. The fractal dimension (*D* = 0.8) is determined from the slope of scattering data from blank resin at low Q (red dashed line). (b) Radial density distribution computed from the scattering intensity plots from the blank resin and antibody bound to MabSelect SuRe 0–80 mg/ml resin. The resin signal is represented in black and evolves toward red with increasing surface concentration [Color figure can be viewed at wileyonlinelibrary.com]

The scattering intensity of the antibody adsorbed to the protein A ligand at the resin surface was measured and plotted in Figure 4a. The black curve corresponds to the MabSelect SuRe resin scattering intensity. Brighter red curves correspond to scattering intensity of MabSelect SuRe resins that have been incubated with a range of antibody concentrations.

The structure factor dimension could be determined by calculating the absolute tangential slope to the low Q range of the scattering intensity of the resin. We found $D_{f}=0.8$, which is in good agreement with the calculated value from the SEM image ($D_{f}=0.74$). The computed fractal dimension from the fractal network of the SEM image matches the estimated fractal dimension we found from the low Q range of the experimental scattering intensities because both are 2D projections. One is an Abel transformation of the 3D pair density distribution and the other a binarized microscopy image of a 2D cut of the resin.

To assess the form factor contribution of the antibody–ligand complex distributed across the resin network at different equilibrium concentrations, we corrected the experimental scattering intensity I(Q) by the fractal structure factor $S(Q)\propto 1/Q^{1.8}$. The resulting P(Q) is seen in Figure 4a, where the experimental data are represented by gray disks and the curve fits with dashed lines. The signal curveture visible along the different Q ranges, is a sign of different surface coverage by the antibody as a function of its bulk concentration.

#### 3.1.5 The appropriate normalization of radial densities

A standard approach for background correction were to subtract the scattering intensity of the antibody‐free resin sample from all of the complement data by $I_{c}(Q)=I(Q)-cI_{b}\left(Q\right)$ and then perform the inverse Fourier transform of the remaining scattering intensity: $p(r)={ℱ}^{-1}(I_{c}\left(Q)\right)$. However, this approach is biased because of the adjustment of factor c.

We propose a modified approach for background correction. First, we corrected all scattering intensities by the structure factor, S(Q), and then computed the radial density distributions: $p(r_{c})={ℱ}^{-1}\left(P(Q)\right)$. Figure 4b shows the radial density distributions, $p\left(r_{c}\right)$, from the scattering intensity profiles of antibody adsorption to MabSelect SuRe at the concentrations displayed in Figure 4a. As in Figure 4a, the curves go from black to red with increasing antibody concentration, with the black curve indicating the radial density distribution of the antibody‐free resin sample. The resin signal (black line) has a maximum at 5–6 nm, which can be interpreted as the minimum radius of an agarose strand. We assume that the antibody molecules bind to the protein A ligands and do not penetrate the cross‐linked agarose strand. Therefore, the radial density distributions for every antibody concentration needs to match until 6 nm, resulting in the normalization of the radial density distributions.

#### 3.1.6 Background‐corrected radial density distribution

**Figure 5:**
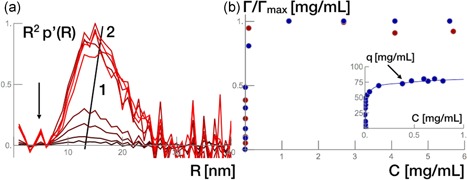
(a) Background‐corrected radial density distributions, p′(R). (b) Surface excess computed from the normalized areas from $R^{2}p\prime \left(R\right)$ as a function of antibody equilibrium concentration (red disks). The adsorbed amount derived from the equilibrium state of the samples before X‐ray exposure (blue disks). The insert shows the experimentally determined adsorption isotherm of antibody adsorption to MabSelect SuRe (blue disks) [Color figure can be viewed at wileyonlinelibrary.com]

To background‐correct the normalized radial density distributions we subtracted the normalized radial density distribution of the resin from the normalized radial density distribution of the signals from samples with antibody bound to protein A. The normalized and background‐corrected pair density distributions are shown in Figure 5a. It shows the radial density distribution of different antibody concentrations. The most distinct feature is the increasing magnitudes with the increasing antibody concentration. The net area of the profiles resemble the surface excess, given by Γ.

#### 3.1.7 Assessing the surface excess

If plotted with respect to the equilibrium bulk concentration of the antibody, the net area of the normalized radial density distribution profiles give a surface excess adsorption isotherm. The normalization of the isotherm was done in respect to the value at the highest concentration (Figure 5b). The normalized surface excess values computed from the radial density distributions are shown in red, and the normalized isotherm derived from the equilibrium state of the samples before X‐ray exposure is given in blue. This match supports the approach of how to assess radial density distributions from the scattering intensity data.

Figure 5b also shows an insert with the experimentally determined adsorption isotherm. It follows the favorable binding rectangular profile characteristic of protein A resins in the antibody uptake. At equilibrium, the data show a second plateau to greater *q*_max_. The data were fitted with a bi‐Langmuir model, as described in the theory section. Results favor a multipoint attachment due to heterogeneous binding sites with a weaker binding of a second antibody molecule to the protein A ligand (Bellot & Condoret, 1993). What needs to be further explored is how in particular does it bind and how is the form of the antibody–protein A complex affected.

#### 3.1.8 A form of antibody–protein A 3D complex by molecular simulation

MabSelect SuRe is a tetrameric protein A chain, thus multipoint attachment is theoretically possible (Gagnon & Nian, 2016; Ghose et al., 2007; Mazzer et al., 2017). Focused research is lacking. We have addressed it on basis of the normalized and background‐corrected radial pair densities at different antibody bulk concentrations.

#### 3.1.9 Antibody–protein A–agarose complex

To visualize SAXS data, and to ease or support their interpretation, we perform reverse Monte Carlo simulations. They are a powerful tool to help the interpretation of the nature of the present data. In this study, we modeled the antibody–protein A complex form by a rigid body approach.

As already mentioned, MabSelect SuRe protein A chain is a recombinant polymer of four units of staphylococcal protein AB domain called the Z‐domain. This Z‐domain is engineered through a point mutation of the B‐domain to give the ligand improved alkaline stability (Ghose et al., 2005). To mimic as closely as possible the chromatographic system involved in this study, we used the crystallographic structure of protein AB domain (PDB: 1BDD) and built a four‐fragment chain.

The model used to represent MabSelect SuRe agarose was kindly provided by Carlo Cavallotti from his group’s publication (Salvalaglio et al., 2009), because they have recently used such a model to predict which amino acid residues contribute the most to IgG binding to protein A. The construct of this agarose model is described in detail in Ref. (Busini et al., 2006). The tetrameric protein A chain was covalently linked to the agarose with an ester bond, and no spacer was introduced.

The final part of the model was the antibodies. We used the crystal structure of an IgG1 (PDB: 1HZH) and bound them through the consensus binding site located between the CH2 and CH3 Fc domains to the Fc binding site of one of the protein AB domains. Deisenhofer has determined a complex of one half of the antibody Fc fragment and one protein AB domain (PDB: 1FC2; Deisenhofer, 1981). Our model matched Deisenhofer’s proposed structure.

#### 3.1.10 Random sampling

After designing the model complex, we ran simulations. We designed a rigid body random walk model. We define systems component: agaroese backbone plus first protein A ligand, adjacent protein A ligands and antibody. All system components were considered rigid entities. Different orientations of the protein A fragments and the antibodies were allowed. Each protein A fragment is considered as a single‐point attachment domain to the antibody. Therefore, in the whole chain there are four potential binding sites; one per fragment.

**Figure 6:**
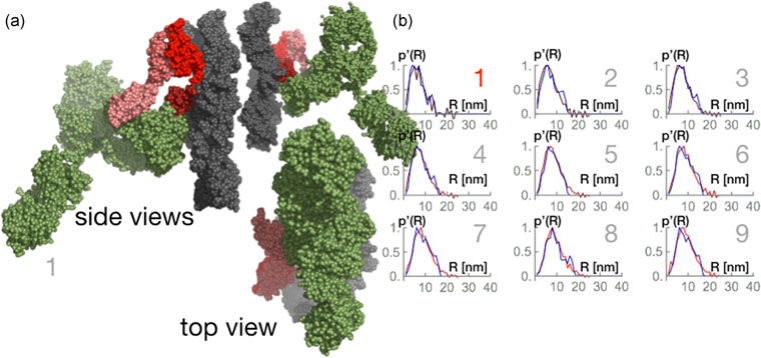
Rigid body models and radial density distributions of 1:1 antibody to protein A stoichiometry. (a) Selected configuration of the complex; the gray bead model indicates the resin; the red bead models mimic the MabSelect SuRe protein A tetrameric chain; the green bead model marks the antibody. (b) Radial density distributions computed from SAXS data (dark red to bright red lines) are compared to radial density distributions (blue line) computed from random walk models. The data enumerated 1–9 correspond to different antibody bulk concentrations, with the correspondence given in the text [Color figure can be viewed at wileyonlinelibrary.com]

The first set of simulations regarded the binding of one antibody molecule to the protein A chain. We simulated a library of at least 10,000 potential forms of antibody in complex with the protein A tetramer ligands and the agarose strand. From the conformations, we assessed the respective radial density distributions. Figure 6a shows the rigid body models of a selected conformation and Figure 6b shows the radial density distributions of 1:1 antibody to protein A chain stoichiometry.

**Figure 7:**
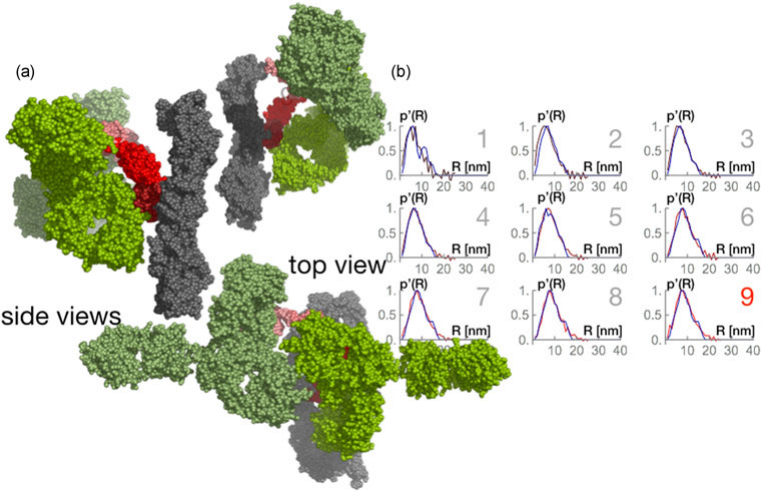
Rigid body models and radial density distributions of 2:1 antibody–Protein A stoichiometry. (a) Selected configuration of the complex; the gray bead model indicates the resin; the red bead models mimic the MabSelect SuRe Protein A tetrameric chain; the green bead model marks the antibody. (b) Radial density distributions computed from SAXS data (dark red to bright red lines) are compared to radial density distributions (blue line) computed from random walk models. The data enumerated 1–9 correspond to different antibody bulk concentrations, with the correspondence given in the text [Color figure can be viewed at wileyonlinelibrary.com]

**Figure 8:**
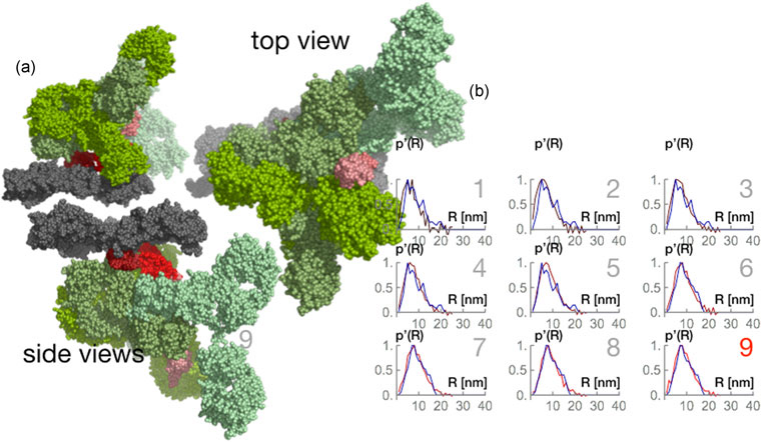
Rigid body models and radial density distributions of 3:1 antibody–protein A stoichiometry. (a) Selected configuration of the complex; the gray bead model indicates the resin; the red bead models mimic the MabSelect SuRe protein A tetrameric chain; the green bead model marks the antibody. (b) Radial density distributions computed from SAXS data (dark red to bright red lines) are compared to radial density distributions (blue line) computed from random walk models. The data enumerated 1–9 correspond to different antibody bulk concentrations, with the correspondence given in the text [Color figure can be viewed at wileyonlinelibrary.com]

With increasing equilibrium concentration (we number them from 1 to 9 in Figure 6b), the best results for 1:1 antibody–protein A stoichiometry show a binding preference for the: fourth (solution 1: *C*= 0.01 mg/ml; *q*= 25.8 mg/ml resin), third (solution 2: *C* = 0.01 mg/ml; *q* = 25.8 mg/ml resin), first (solution 3: *C* = 0.01 mg/ml; *q*= 25.8 mg/ml resin), first (solution 4: *C* = 0.01 mg/ml; *q* = 38.7 mg/ml resin), third (solution 5: *C* = 0.1 mg/ml; *q* = 64.5 mg/ml resin), third (solution 6: *C* = 1.2 mg/ml; *q* = 80.0 mg/ml resin), third (solution 7: *C* = 2.7 mg/ml; *q* = 80.0 mg/ml resin), fourth (solution 8: *C* = 4.7 mg/ml; *q* = 80.0 mg/ml resin), and third (solution 9: *C* = 5.6 mg/ml; *q* = 80.0 mg/ml resin) domain counting from the agarose surface. The obtained preferential binding is speculative as it does not take into account any energy minimization. Simulations indicate that at low bulk concentrations and very low surface concentrations (solutions 1 and 2) the antibody binds to the outermost ligands (fourth and third) but finds itself in the proximity of the first ligand. Engineered protein A in commercial media has a tentacle form and the chain could be extended in the surface (Gagnon & Nian, 2016). We have implemented the possibility for the protein A chain for a loop‐like conformation (see its form in Figures 6, 7, or 8). Within our random walk model the protein A chain is flexible and a transfer from the outermost to the innermost ligand seems plausible. Biologically, antibody dual‐site binding to protein A is possible (Gagnon & Nian, 2016). With increasing surface concentration but still at low equilibrium concentration (solutions 3 and 4), the first ligand is the most favored. At elevated concentrations (solutions 5–9) the outermost become favored again. At these concentrations, the radial densities of the simulated configurations lack the tailing we find in the experimental data, as seen in Figure 6b. A second antibody molecule is added to recover the partiuclar tailing.

Following a 2:1 stoichiometry, we attached two antibody molecules to every possible combination of B fragments and allowed every possible orientation. Figure 7a shows possible orientations of two antibody molecules bound to the inner and outermost fragments in the protein A. Again, the radial density distributions of these models were determined and scanned for similarity to radial densities computed from the experimental SAXS data. Figure 7b shows the radial density distributions of this 2:1 stoichiometry.

Antibody molecules bound to the two outermost fragments returned the best radial density distributions, matching the distribution at high antibody concentration provided by SAXS. It could be assumed that the steric hindrance from the agarose would be greater in comparison to the resulting hindrance of the close proximity of another antibody molecule. The radial density distributions at this moment showed a maximum detected relative distance at around 21 nm. This value could correspond to the largest possible distance between the two antibody molecules (approximately the sum of two hydrodynamic radii) or the distance from the most external antibody to the agarose.

These modeled data support the isotherm prediction. At saturation, more than one antibody molecule can bind to the protein A ligands with the support of binding heterogeneity proposed by bi‐Langmuir isotherm model. Data based on equilibrium binding capacities definitely support the idea that two antibody molecules could be bound to the MabSelect SuRe ligand. This was already suggested by other authors with protein A in solution studies (Ghose et al., 2007) and with neutron reflectivity studies with protein A attached to silica (Mazzer et al., 2017).

Finally, we ran models with three antibody molecules bound to different protein A fragments within the same chain. The configurations computed are densely packed. Figure 8a shows a selected configuration of these models. Figure 8b shows the radial density distributions of this stoichiometry overlapped with the experimental data.

We are argue that a 3:1 stoichiometry possibility could be excluded because of the steric effects. We would need to consider all atomistic pairwise interactions to argue their feasibility. In the current study, we limited ourselves to geometrical considerations.

## 4 CONCLUSIONS

In this study we experimentally assessed radial density distributions and, on basis of this experimental value, hypothesized possible antibody–protein A forms and configurations in a chromatographic resin.

We used small‐angle X‐ray scattering as an experimental method to model and speculate on the 3D form of antibody in solution and after binding to tetrameric staphylococcal protein A in MabSelect SuRe. We compared the experimentally assessed radial density distribution with ones computed from molecular simulations. Computational models were restricted to crystallographic data and to data derived from the molecular dynamic simulations.

We reason that the antibodies bind to the protein A ligand at different stoichiometries because of the existence of heterogeneous binding sites. At low antibody concentrations (<40 mg/ml resin) we argue that the probable binding stoichiometry is 1:1, whereas at higher concentrations (>40 mg/ml resin) a 2:1 stoichiometry is favored. At low concentrations, and assuming a 1:1 stoichiometry, the random walk models point toward configurations where the antibody binds at the outermost ligands at very low and at high concentrations and in the perpendicular form in respect to the surface. At 2:1 stoichiometry, we favor propeller‐like configurations of the immobilized antibodies, which are more preferentially bound to the first and fourth ligand. From our data, a 3:1 stoichiometry, albeit theoretically possible, is excluded here because of the steric effects.

We are convinced that our study, in which we outlined how to rationally assess 3D forms of the antibody–protein A complexes at different antibody concentrations next to a resin surface, will trigger the rational design of this technology of high industrial relevance. Our experimental design could be potentially used to investigate molecule binding on other chromatographic systems in terms of stoichiometry, binding configurations, and distal spacing. Therefore, it can be implemented as a monitoring tool in industrial applications where it is necessary to purify large amounts of the product while obeying to certain Quality by Design parameters.

## CONFLICTS OF INTEREST

The authors declare that there are no conflicts of interest.
